# Implementation of the World Health Organization Regional Office for Africa Stepwise Laboratory Quality Improvement Process Towards Accreditation

**DOI:** 10.4102/ajlm.v5i1.280

**Published:** 2016-05-20

**Authors:** Jean-Bosco Ndihokubwayo, Talkmore Maruta, Nqobile Ndlovu, Sikhulile Moyo, Ali Ahmed Yahaya, Sheick Oumar Coulibaly, Francis Kasolo, David Turgeon, Angelii P. Abrol

**Affiliations:** 1 World Health Organization, Regional Office for Africa, Brazzaville, Congo; 2 African Society for Laboratory Medicine, Addis Ababa, Ethiopia; 3 Botswana-Harvard AIDS Institute Partnerships, Gaborone, Botswana; 4 United States Centers for Disease Control and Prevention, Center for Global Health, Division of Global HIV and Tuberculosis, Atlanta, Georgia, United States

## Abstract

**Background:**

The increase in disease burden has continued to weigh upon health systems in Africa. The role of the laboratory has become increasingly critical in the improvement of health for diagnosis, management and treatment of diseases. In response, the World Health Organization Regional Office for Africa (WHO AFRO) and its partners created the WHO AFRO Stepwise Laboratory (Quality) Improvement Process Towards Accreditation (SLIPTA) program.

**SLIPTA implementation process:**

WHO AFRO defined a governance structure with roles and responsibilities for six main stakeholders. Laboratories were evaluated by auditors trained and certified by the African Society for Laboratory Medicine. Laboratory performance was measured using the WHO AFRO SLIPTA scoring checklist and recognition certificates rated with 1–5 stars were issued.

**Preliminary results:**

By March 2015, 27 of the 47 (57%) WHO AFRO member states had appointed a SLIPTA focal point and 14 Ministers of Health had endorsed SLIPTA as the desired programme for continuous quality improvement. Ninety-eight auditors from 17 African countries, competent in the Portuguese (3), French (12) and English (83) languages, were trained and certified. The mean score for the 159 laboratories audited between May 2013 and March 2015 was 69% (median 70%; SD 11.5; interquartile range 62–77). Of these audited laboratories, 70% achieved 55% compliance or higher (2 or more stars) and 1% scored at least 95% (5 stars). The lowest scoring sections of the WHO AFRO SLIPTA checklist were sections 6 (Internal Audit) and 10 (Corrective Action), which both had mean scores below 50%.

**Conclusion:**

The WHO AFRO SLIPTA is a process that countries with limited resources can adopt for effective implementation of quality management systems. Political commitment, ownership and investment in continuous quality improvement are integral components of the process.

## Introduction

The burden of disease, especially infectious disease, continues to weigh heavily upon health systems in Africa, where diseases such as HIV, malaria, tuberculosis, acute respiratory infections and diarrhoea continue to have high mortality rates.^1^ A high burden of disease is devastating in developing economies where it adversely affects all components of societal development, including income, health, and education.

The role of the laboratory is increasingly recognised as critical in the overall improvement of countries’ health systems.^2^ Medical and public health laboratories play a central role in the continuum of healthcare from diagnosis to treatment, management of patients and surveillance of diseases.^2,3,4^ Without a definitive diagnosis from the laboratory, treatment is often based on syndromic patient management, in which symptoms that are often unspecific are used for decision-making, potentially leading to incorrect treatment.^5^ This can often lead to extended hospital stays, unnecessary admissions, loss of quality of life, deaths, irrational use of antimicrobial drugs and economic burden on families.^6^

Realising the central role of the laboratory, increased funding from global health partners, such as the United Nations’ Global Fund to Fight HIV, Tuberculosis and Malaria, the United States President’s Emergency Fund for AIDS Relief, the World Bank and the Bill & Melinda Gates Foundation, has targeted the strengthening of laboratory systems.^2,3,4^ To capture this demonstrated impact of laboratories on overall patient outcomes, satisfaction levels, hospital stays and cost of care, reliable information in the form of quality laboratory results is required. To produce quality results consistently, laboratories need to implement and maintain continuous quality improvement systems that ensure policies, procedures, organisational and technical requirements are established as part of their quality management system.^7^ Attaining accreditation is one means of independently confirming the existence of a quality management system^4^ and that the laboratory has the competence to perform high-quality testing.^8^

The number of laboratories in Africa with internationally-recognised accreditation status has been very low, with figures below 400 reported as of 2014.^9,10^ Although the exact total number of medical laboratories in Africa is not known, Uganda alone had 1234 government laboratories in 2007.^9^ This is a clear indication of how few laboratories in Africa have successfully implemented quality management systems with the goal of international accreditation. In 2014, Schroeder et al. reported 380 accredited laboratories in sub-Saharan Africa, 345 (91%) of which were located in South Africa and 37 of the 49 of these countries had no laboratories accredited to international standards as of May 2013.^10^

Countries in the African region have long recognised the need to strengthen laboratory systems, dating back to the Maputo Declaration in 2008.^11^ Over the following years, the World Health Organization (WHO), in collaboration with its Member States, made further commitments to remove identified barriers linked to poor laboratory systems, including poor quality management systems. This notably included the September 2008 resolution AFR/RC58/R2 on Public Health Laboratory Strengthening adopted during the 58th session of the WHO Regional Committee in Yaoundé, Cameroon^12^ and the 2008 Joint WHO-United States Centers for Disease Control and Prevention (CDC) conference on laboratory quality systems in Lyon, France, where implementation of laboratory quality management systems in a staged approach was first recognised and endorsed by partners.^13^ Recognising the need for a strong laboratory system in the efforts to maximise gains made through investments in health systems, these commitments by the governments of Africa were critical in solidifying the much-needed political commitment to drive the agenda for laboratory system strengthening.

A key recommendation from the 2008 Joint WHO-CDC meeting was for the WHO to develop potential models of accreditation preparedness for adaptation and use by Member States.^13^ The WHO Regional Office for Africa (WHO AFRO), the United States CDC, the Clinton Health Access Initiative, the American Society for Clinical Pathology and other partners launched the first version of the WHO AFRO stepwise accreditation preparedness process in Kigali, Rwanda, in 2009.^14^ In 2011, in Nairobi, Kenya, it was renamed the WHO AFRO Stepwise Laboratory (Quality) Improvement Process Towards Accreditation (SLIPTA).^15^ SLIPTA is a framework for a stepwise quality improvement process for clinical, medical and public health laboratories in developing countries aimed at achieving the requirements of the International Organization for Standardization (ISO) 15189 clinical laboratory standard.^15^ The policy guidance and checklist were published by WHO AFRO in July 2013, in English, French and Portuguese.^15^ The revised SLIPTA checklist replaced the WHO AFRO Accreditation Checklist and was expanded to include 111 items worth a total of 258 points. The policy document provides guidance for implementation and describes the key elements, governance structure and key stakeholder roles and responsibilities. It defined key terms such as standard, certification, accreditation, standardisation bodies – and their respective applicability – and broadened ‘accreditation bodies’ to include national, regional and international bodies. The term ‘assessment’ was replaced by ‘audit’ to differentiate the stepwise recognition process from an assessment conducted by an accrediting body. The intent of SLIPTA was to embed the continuous improvement culture in laboratories whilst providing an avenue for laboratories to eventually meet the standards required for national, regional, or international accreditation.^15^ Concurrently, the Strengthening Laboratory Management Toward Accreditation (SLMTA) programme was launched. SLMTA is a hands-on, activity-based training programme designed to support the implementation of quality management systems in laboratories seeking recognition through SLIPTA.

Endorsed by the Ministerial Call for Action at the 1st International African Society for Laboratory Medicine (ASLM) Conference in December 2012,^16^ the *ASLM2020: Strategies and Vision to Strengthen Public Health Laboratory Medicine in Africa* recognised the challenges in public health laboratories and inspired the region to achieve excellence in laboratories.^17^ Six Ministers of Health initially endorsed this call for action. It emphasised ASLM’s role in addressing these challenges through collaborative partnerships with governments, national, regional and international partners and organisations, the private sector and other stakeholders. The ASLM2020 goal for laboratory accreditation includes enrolment of 2500 laboratories in SLIPTA and 250 public laboratories accredited by international standards by the year 2020.^17^

In this article, we describe the implementation of the WHO AFRO SLIPTA programme and progress to date in 18 countries.

### Governance structure

**FIGURE 1:**
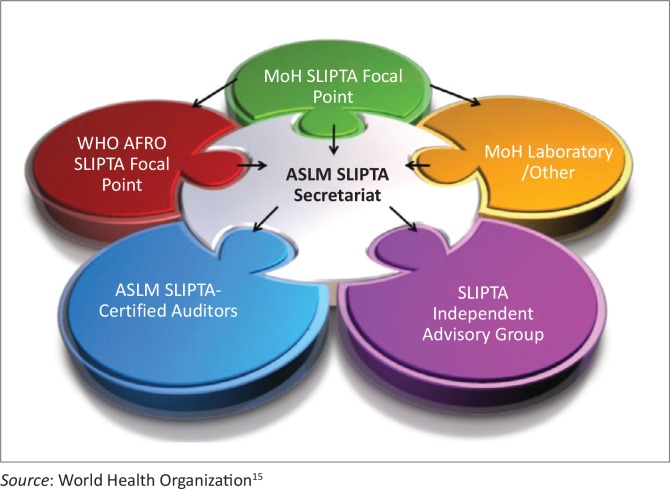
The WHO AFRO SLIPTA governance structure.

The WHO AFRO SLIPTA guidance document defines the SLIPTA governance structure with six main stakeholders^15^ (Figure 1) and WHO AFRO’s role of setting the norms and standards and implementing programmes through partnerships. The Independent Evaluating Group, established through a memorandum of understanding with WHO AFRO, implemented SLIPTA by first identifying a secretariat and second, establishing a SLIPTA Independent Advisory Group, comprising regional and international experts in laboratory quality management systems and accreditation who oversee the coordination and implementation of the process.

The secretariat, identified as ASLM in 2011, coordinates the implementation of SLIPTA in close collaboration with Ministries of Health, CDC and other regional and local development partners.^15^ The secretariat works with the WHO AFRO SLIPTA focal points.

## SLIPTA implementation

Implementation of SLIPTA was defined by WHO AFRO in its SLIPTA guidance document of 2011,^15^ which provided the policy direction for all stakeholders and the tools to be used in the application, audit and recognition processes, as described below.

### Country SLIPTA focal points

The guidance document provided for the nomination of a national SLIPTA focal point by each member state. In October 2012, WHO AFRO issued a memorandum endorsing the SLIPTA policy, checklist technical documents and implementation of SLIPTA in the region and requested Member states to designate a national focal point for WHO AFRO SLIPTA. It stated that SLIPTA would be aligned with the Maputo Declaration, Resolution AFR/RC58/R2 issued in Yaoundé, the initial launch of SLIPTA in Kigali and the Millennium Development Goals.^18^ The country SLIPTA focal points play a governmental support role by coordinating all in-country SLIPTA implementation activities. They oversee the application process for laboratories enrolling in SLIPTA and work with laboratories, ASLM and local implementing partners to organise audits and to implement post-audit improvement plans. In collaboration with ASLM, SLIPTA focal points also oversee the selection, training and certification of in-country SLIPTA auditors. By March 2015, 27 of the 47 WHO AFRO Member States had nominated a national SLIPTA focal point, including Algeria, Angola, Burkina Faso, Burundi, Tanzania, Mozambique, South Africa, Uganda, Kenya, Zambia, Malawi, Botswana, Namibia, Cameroon, Senegal, Côte d’Ivoire, Chad, Swaziland, Lesotho, Nigeria, Mali, Zimbabwe, Cape Verde, São Tomé and Principe, Gambia, Benin and Togo.

### The WHO AFRO SLIPTA checklist

The WHO AFRO SLIPTA scoring checklist was revised in 2011, two years after the initial launch of the programme, and is based on international standards, including the ISO 15189:2007 standard^19^ and the Clinical Laboratory Standards Institute guideline GP26-A3.^20^ The checklist has 111 items organised into 12 quality systems essentials with a maximum score of 258 points (Table 1), allowing for quantitative measurement of progress in the implementation and adherence to accreditation requirements for quality and competency. The checklist was revised and published by WHO AFRO in 2015 to align with the ISO 15189:2012 standard.

**TABLE 1:** The WHO AFRO SLIPTA checklist covering the 12 quality system essentials, the points allocated to each section and the overall star rating system.

WHO AFRO SLIPTA Checklist Audit Score Sheet (Section)	Total Points
Section 1: Documents & Records	25
Section 2: Management Reviews	17
Section 3: Organization & Personnel	20
Section 4: Client Management & Customer Service	8
Section 5: Equipment	30
Section 6: Internal Audit	10
Section 7: Purchasing and Inventory	30
Section 8: Process Control and Internal & External Quality Audit	33
Section 9: Information Management	18
Section 10: Corrective Action	12
Section 11: Occurrence/Incident Management & Process Improvement	12
Section 12: Facilities and Safety	43

**Total Score**	**258**

*Source*: World Health Organization^15^

0 stars, (0–142 pts), < 55%; 1 star, (143–165 pts), 55% - 64%; 2 stars, (166–191 pts), 65% - 74%; 3 stars, (192–217 pts), 75% - 84%; 4 stars, (218–243 pts), 85% - 94% 5 stars, (244–258 pts), ≥ 95%.

WHO AFRO, World Health Organization Regional Office for Africa; SLIPTA, Stepwise Laboratory (Quality) Improvement Process Towards Accreditation.

The scoring checklist allows for laboratory performance to be rated using a scale of 0 to 5 stars with each star level associated with the laboratory’s total score on the audit as follows: 0–142 points = 0 stars, 143–165 points = 1 star, 166–191 points = 2 stars; 192–217 points = 3 stars, 218–243 points = 4 stars and 244–258 points = 5 stars. At the end of the audit, each laboratory that achieves a star level of one star or higher is issued a corresponding star level certificate that is valid for two years (Table 1). Progress up the star scale denotes increased compliance with the WHO AFRO SLIPTA checklist aligned with international standards.

### Laboratory enrolment

The country SLIPTA focal point coordinates the application process with ASLM for enrolment into SLIPTA and is expected to prioritise laboratories as defined by their country’s strategic plans and tiered laboratory network. Minimum criteria for enrolment for a laboratory includes at least one star (at least 143 points) achieved from an internal audit, a completed application form, an approved quality manual or equivalent, an organisational structure diagram or organogram and an application fee. Laboratory applications denote the type of laboratory as public, private or research and the level of the laboratory as national, reference, regional or district, depending on the country.

All applications submitted through the country SLIPTA focal point to the ASLM secretariat are reviewed and approved for enrolment if all criteria are met. Audit logistics, such as selection and deployment of auditors, are coordinated by ASLM.

### Auditor training, selection and deployment

The WHO AFRO SLIPTA audits are conducted by ASLM-certified auditors. Auditor training consists of a five-day standardised training curriculum plus a practicum, which comprises the successful completion of three to five SLIPTA audits under the observation of a lead auditor. The lead auditor evaluates the auditor trainee’s competency based on standard evaluation criteria that include: rating the trainee on his/her understanding of the WHO AFRO SLIPTA process, auditing skills; and understanding of the WHO AFRO SLIPTA checklist and the ISO 15189 standard.

Three master ASLM trainers, initially trained by WHO AFRO and the Southern African Development Community Accreditation Service, provided the initial training and certification of auditors. Subsequently, capacity to conduct auditor training has been built by other collaborating partners, including the National Health Services of South Africa, A Global Healthcare Public Foundation of Uganda and individual master trainers from the ministries of health.

Each audit team, led by a lead auditor, comprises at least two certified auditors, depending on the size and scope of the testing of the laboratory. Team composition is guided by careful review of the laboratory application documents to make sure the selected audit team competencies match the laboratory’s scope of testing. When multiple laboratory teams are deployed concurrently in a country, ASLM appoints an overall lead auditor to coordinate all audits and submission of audit reports to the ASLM secretariat.

### Laboratory audits

The standard audit process starts with an opening meeting, the audit on day one and a feedback meeting on day two. The SLIPTA audit follows a horizontal approach whereby the auditors review quality and technical records, ask questions of the laboratory staff and observe laboratory practices on the day of the audit. Unique to SLIPTA, the auditor has two key responsibilities: (1) identification of areas of non-conformity clearly documented using objective evidence and, more importantly, (2) providing on-site technical assistance and recommendations for closing identified gaps, differentiating it from a standard accreditation assessment.

At the end of the audit, usually on day one, the audit teams discuss their findings, agree on scores and develop a draft audit report with documented non-conformities and recommendations. The lead auditor submits the draft audit report to the laboratory for review prior to the debrief meeting on day two. The final report is agreed upon by the laboratory and the audit team by the end of the debrief meeting before submission to the ASLM secretariat. The final report is supported by a form agreeing to the report content and signed by the laboratory representative.

Key to the SLIPTA process is advocacy for laboratory system strengthening in the target countries. Built into the SLIPTA audit process are opportunities for advocating for the laboratories where each audit team must meet hospital management before and after each audit. Depending on country context, this may also include provincial or district management teams. When multiple laboratories are audited, debrief is held with high-level ministry of health officials, WHO country representatives, laboratory directorate, in-country implementation partners, laboratory associations, and managers of audited laboratories. During the debrief session, the auditors provide feedback on the overall performance of the audited laboratories and advocate for implementation of corrective actions identified during the audit.

### Star recognition

Star determination and the issuance of the certificate of recognition is recommended by the Independent Advisory Group based on the audit reports. After review, the Independent Advisory Group recommends the star level to the ASLM secretariat, who issues the certificate of recognition; these certificates are valid for two years.

The laboratory is expected to apply for another audit six months before the expiration of the current recognition certificate as part of the re-audit process. At this point, the laboratory, country focal point and local implementing partners would have worked to address the gaps identified in the previous audit. It is through this process of incremental implementation of quality requirements that the laboratory progresses over the 0- to 5-star rating until it can be recommended to apply for international ISO 15189 accreditation.

## Preliminary outcomes

**FIGURE 2:**
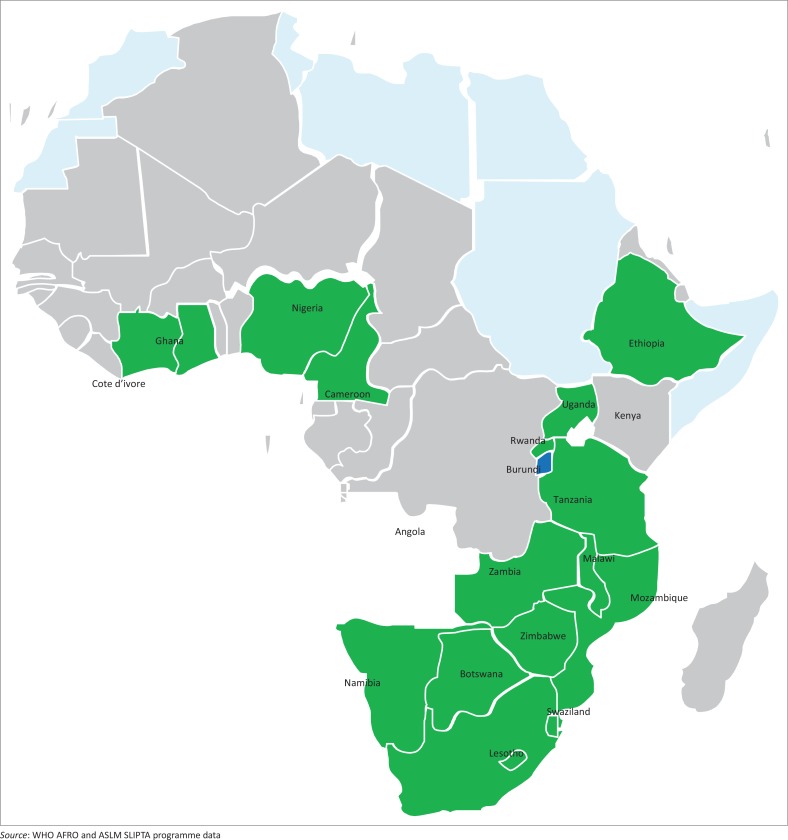
Map of the 18 WHO AFRO member states that were SLIPTA-audited from May 2013 to March 2015.

**FIGURE 3:**
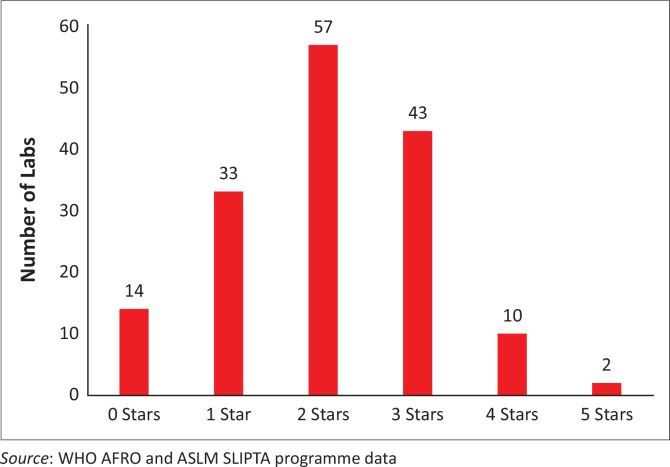
Distribution of stars across the 159 SLIPTA-audited laboratories, May 2013 to March 2015.

By March 2015, 27 of 47 (57%) WHO AFRO member states had identified and appointed a SLIPTA focal point to coordinate their in-country SLIPTA activities. At the ASLM 2012 bi-annual conference in December 2012, six Ministers of Health endorsed SLIPTA as the framework for strengthening laboratory services in their respective countries;^16^ an additional eight Ministers of Health have endorsed SLIPTA since then, for a total of 14 Ministerial endorsements. A total of 98 auditors from 17 African countries, with competencies in the Portuguese (3), French (12) and English (83) languages, have been trained and certified and conducted the audits (Table 2). Between May 2013 and March 2015, 159 laboratories from 18 of the 27 African countries with SLIPTA focal points were audited (Figure 2). Of these 159 laboratories, 145 (91%) achieved at least one star, the majority achieved two stars (57 laboratories) and 2 (1%) received five stars on the SLIPTA checklist (Figure 3).

**TABLE 2:** Summary of laboratories audited through the SLIPTA process and certified SLIPTA auditors by country, May 2013 to March 2015.

Country	Number of audited laboratories			Number of certified auditors

	Public	Private	Total	
Angola	3	0	3	0
Botswana	11	0	11	7
Burkina Faso	0	0	0	1
Cameroon	8	1	9	7
Côte d’Ivoire	4	0	4	4
Ethiopia	9	0	9	6
Ghana	14	0	14	7
Kenya	0	0	0	1
Lesotho	5	0	5	2
Malawi	11	1	12	4
Mozambique	4	0	4	3
Namibia	3	1	4	0
Nigeria	16	0	16	11
Rwanda	2	0	2	0
South Africa	14	0	14	9
Swaziland	9	0	9	6
Tanzania	22	1	23	13
Uganda	8	0	8	9
Zambia	1	1	2	2
Zimbabwe	10	0	10	3
ASLM	N/A	N/A	N/A	3

**Total**	**154**	**5**	**159**	**98**

SLIPTA, Stepwise Laboratory (Quality) Improvement Process Towards Accreditation; ASLM, African Society of Laboratory Medicine; N/A, not applicable.

The mean score for all 159 laboratories was 69% (median, 70%; SD, 11.5; interquartile range [IQR], 62–77) (Table 3). SLIPTA checklist sections 4 (Client Management), 9 (Information Management) and 12 (Facilities and Safety) had mean scores of at least 80%, whereas for checklist sections 6 (Internal Audit) and 10 (Corrective Action), the mean scores were below 50%. The greatest variability, as shown by the IQR, was observed for checklist sections 2 (Management Reviews; IQR, 41–71), 6 (Internal Audit; IQR, 20–60) and 11 (Occurrence Management; IQR, 33–92).

**TABLE 3:** Summary of performance of the 159 laboratories across 12 sections of the WHO AFRO SLIPTA checklist.

Section	Quality System Essential	Median (%)	25th–75th Interquartile Range	Mean (%)	95% CI
Section 1	Documents and Records	56	52–68	59	56–61
Section 2	Management Reviews	53	41–71	54	50–57
Section 3	Organization and Personnel	70	60–80	72	70–74
Section 4	Client Management and Customer Service	88	75–100	82	80–85
Section 5	Equipment	73	63–80	71	69–73
Section 6	Internal Audit	20	20–60	42	37–47
Section 7	Purchasing and Inventory	80	70–90	77	74–79
Section 8	Process Control and Internal and External Quality Assessment	70	61–79	69	67–72
Section 9	Information Management	83	72–94	80	78–83
Section 10	Corrective Action	42	33–58	44	41–48
Section 11	Occurrence/Incident Management and Process Improvement	58	33–92	57	52–62
Section 12	Facilities and Safety	86	74–91	83	81–85

**Final percentage score**		**70**	**62–77**	**69**	**67–71**

*Source*: World Health Organization^15^

WHO AFRO, World Health Organization Regional Office for Africa; SLIPTA, Stepwise Laboratory (Quality) Improvement Process Towards Accreditation; CI, confidence interval.

Two SLIPTA-audited laboratories, the National Tuberculosis Reference Laboratory in Maputo, Mozambique and the Princess Marina Hospital Laboratory in Gaborone, Botswana, have since attained international ISO 15189 accreditation. Four laboratories in Cameroon have since applied to the South Africa National Accreditation System and await assessment.

## Discussion

Based on these preliminary results, the WHO AFRO SLIPTA programme has been met with positive responses regionally, as observed by its rapid expansion over the past three years of implementation and endorsement by Ministers of Health. This is the first large-scale, regional, standardised approach of its kind to assess laboratories’ preparedness toward international accreditation standards using an external auditing process, with the premise that a star score in one country would be comparable to that of another country across the continent. The audit findings can be used as a roadmap for countries to achieve accreditation through measurable steps of continuous quality improvement by pinpointing specific areas for improvement as part of strategic planning. Tools, such as SLMTA, can be used to improve the identified areas where corrective actions are required, as part of the laboratory’s continuous quality improvement.

Similar stepwise accreditation preparedness approaches have been adopted in other regions globally to encourage continuous laboratory quality improvement. These include the Accreditation Program of Clinical Laboratories established by Argentina in 1994;^21^ the Thailand Medical Technology Council national accreditation program of 2002^22^ and the Korean Laboratory Accreditation Program of 1999.^23^ In all cases, there was significant progress in the implementation of quality management systems toward accreditation.^21,22,23^ Similarly, SLIPTA was adopted to address quality improvement incrementally toward achieving accreditation requirements in the resource-limited settings of Africa. It recognises and rewards efforts made toward meeting accreditation requirements as measured by the achieved percentage of compliance and star levels. This regional approach should transition into national governance as countries develop their national capacity and establish their own accreditation bodies, similar to those in Argentina, Thailand and Korea.

On average, laboratories performed poorly (mean: < 50%) on the Internal Audit and Corrective Action sections of the SLIPTA checklist. These two quality system essentials are linked, in that they form part of the continual improvement activities inherent in a quality management system. For all areas identified as non-conforming, a root cause analysis followed by corrective actions and preventative actions must be performed. Hence, if laboratories do not perform well in the process of non-conformity identification, root cause analysis, preventative actions and monitoring of the effectiveness of corrective and preventative actions, they are not likely to perform well on the internal audit process.

On the other hand, performance in the areas of Client Management, Inventory Control and Information Management were all above 50%, indicating better progress. Notably, almost all laboratories had conducted a customer survey, which forms the basis for understanding the needs of its clients. The WHO AFRO SLIPTA checklist primarily evaluates how inventory is managed once received in the laboratory with a few questions that evaluate the entire procurement system. For laboratories that did not have an electronic-based information system, a paper-based system was always available, which the ISO 15189 standard recognises as acceptable; hence, the better performance in this area.

The results also showed that countries are at very different levels of capacity for implementation of the SLIPTA process. In particular, the Francophone and Lusophone countries are not well covered. This could be because most of the countries covered by the SLIPTA audits to date have benefitted from programmes such as the United States President’s Emergency Plan for AIDS Relief.

Generally, at the time of the audits, most of the laboratories had not surpassed the 3-star rating and only two laboratories had achieved five stars. This implies that although the SLIPTA process has been widely accepted by most countries and notable improvements made, the majority of laboratories have yet to reach international accreditation readiness. To reach their goals, country strategic planning with committed resources for infrastructure, human resources and training in laboratory quality improvement and structured laboratory mentoring are all part of continuous quality improvement and accreditation preparedness.

### Recommendations

Based on the preliminary audit results from 159 laboratories over three years, the majority of laboratories reached two stars. The adoption of the SLIPTA scheme is a viable option for countries with limited resources to guide their path of accreditation preparedness. SLIPTA audit findings equip countries to identify and address specific areas of improvement in the laboratories. This will empower the countries to make informed decisions as part of national planning through smart investments. To support the scale-up of SLIPTA, advocacy and coordination is critical; all 47 Member states should have a SLIPTA focal point to guide implementation of the national SLIPTA strategy as part of a continuous improvement process. Member states are encouraged to integrate SLIPTA and resources for implementation into their national laboratory policies and strategic plans, invest sustained financial and human resources to strengthen the laboratory system and the country’s auditing capacity and strengthen the countries’ national capacity and governance to oversee SLIPTA under a national accreditation body.

We recommend further analysis on the possible barriers to the implementation of quality management systems in Africa, whether countries have used the SLPITA audit findings to improve their laboratories and how SLIPTA has helped improve the laboratories from initial audit to follow-up audits. The internal audit and corrective actions quality system essentials need further review to better understand the challenges laboratories face, for example, which specific checklist questions are laboratories failing to address. An understanding of the profile of laboratories and their performance would assist in understanding the greater variability observed in sections 2 (Management Reviews) and 11 (Occurrence Management).

### Conclusion

Based on the results, the WHO AFRO SLIPTA is a process that countries with limited resources can adopt for effective implementation of quality management systems. Political commitment, ownership and investment in continuous quality improvement are integral components of the process.
